# Enhancing antibody-antigen interaction prediction with atomic flexibility

**DOI:** 10.1371/journal.pcbi.1013576

**Published:** 2025-10-13

**Authors:** Sara Joubbi, Alessio Micheli, Paolo Milazzo, Giorgio Ciano, Stéphane M. Gagné, Pietro Liò, Duccio Medini, Giuseppe Maccari

**Affiliations:** 1 Department of Computer Science, University of Pisa, Pisa, Italy; 2 Data Science for Health Lab, Fondazione Toscana Life Sciences, Siena, Italy; 3 Department of Biochemistry, Microbiology and Bioinformatics, Université Laval, Québec, Canada; 4 Department of Computer Science and Technology, University of Cambridge, Cambridge, United Kingdom; Tel Aviv University, ISRAEL

## Abstract

Antibodies are indispensable components of the immune system, known for their specific binding to antigens. Beyond their natural immunological functions, they are fundamental in developing vaccines and therapeutic interventions for infectious diseases. The complex architecture of antibodies, particularly their variable regions responsible for antigen recognition, presents significant challenges for computational modeling. Recent advancements in deep learning have markedly improved protein structure prediction; however, accurately modeling antibody-antigen (Ab-Ag) interactions remains challenging due to the inherent flexibility of antibodies and the dynamic nature of binding processes. In this study, we examine the use of predicted Local Distance Difference Test (pLDDT) scores as indicators of residue and side-chain flexibility to model Ab-Ag interactions through a fingerprint-based approach. We demonstrate the significance of flexibility in different antibody-specific tasks, enhancing the predictive accuracy of Ab-Ag interaction models by 4%, resulting in an AUC-ROC of 92%. In addition, we showcase state-of-the-art performance in paratope prediction. These results emphasize the importance of accounting for conformational flexibility in modeling antibody-antigen interactions and show that pLDDT can serve as a coarse proxy for these dynamic features. By optimizing antibody flexibility using pLDDT, they can be engineered to improve affinity or breadth for a specific target. This approach is particularly beneficial for addressing highly variable pathogens like HIV and SARS-CoV-2, as greater flexibility enhances tolerance to sequence variations in target antigens.

## Author summary

Antibodies are crucial immune molecules that bind to antigens with high specificity, playing a central role in host defense, vaccine development, and therapeutic interventions. However, accurately modeling antibody-antigen (Ab-Ag) interactions presents significant challenges due to the intrinsic structural flexibility of antibodies, particularly in the complementarity-determining region H3 (CDRH3) and associated side chains. This study examines the impact of flexibility on antibody-antigen interactions by employing a fingerprint-based approach that incorporates ESMFold confidence scores as a proxy for antibody flexibility. We achieved a 4% improvement in predictive accuracy for Ab-Ag interactions, resulting in an AUC-ROC of 92% and demonstrated state-of-the-art performance in the prediction of paratopes. These findings underscore the importance of incorporating pLDDT in the prediction and design of antibody-antigen interactions.

## Introduction

**Fig 1:**
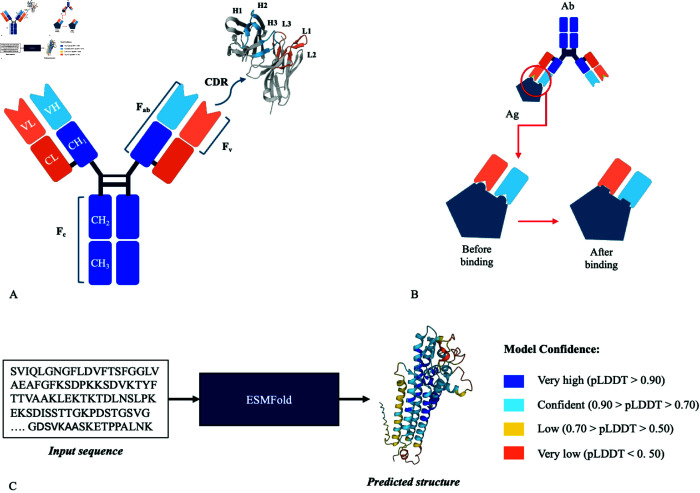
Antibody structure, flexibility, and pLDDT. A) The heavy chain (blue) and light chain (orange) of the antibody are shown, with an inset displaying labeled CDR loops (PDB 3IY3). The Fab region includes the variable and part of the constant domains, while the Fv consists solely of the variable domains (VH and VL); the Fc comprises the remaining constant domains; B) An accompanying illustration demonstrates the dynamic flexibility of the antibody following antigen binding; C) ESMFold generates a 3D structure from a protein sequence, assigning pLDDT scores to atoms. High scores indicate accurate folding and low flexibility, while low scores suggest uncertainty and increased flexibility.

Antibody-antigen interactions are critical for immune defense, neutralizing pathogens, and marking antigens for clearance by immune cells[1]. Their high specificity underlies both natural immunity and the development of targeted therapies for cancer, autoimmune, and infectious diseases [2]. Structurally, antibodies are tetrameric molecules consisting of two heavy (H) and two light (L) chains. The variable (V) regions, containing the complementarity-determining regions (CDRs), mediate antigen binding, while the constant (C) regions provide structural integrity and effector functions via interchain disulfide bonds (see Fig 1A) [3].

The increase in antibody-based therapies is demonstrated by the growing number of FDA approvals, clinical trials, and patent filings [4]. Market forecasts predict the global antibody market will surpass $400 billion by 2028, growing at approximately 14.1% annually [3,5]. Traditional discovery methods, like phage display and animal immunization, are now being complemented by computational approaches that streamline design and optimization, reducing development time and cost while boosting the effectiveness of next-generation immunotherapies.

Deep learning has significantly impacted structural biology. Tools such as AlphaFold2 (AF2) [6] and AlphaFold3 (AF3) [7] have set new benchmarks for predicting protein structures and docking molecules from amino acid sequences. However, these models that rely on multiple sequence alignments (MSAs) do not accurately capture the structure and orientation of Ab–Ag interactions. Although typically classified as protein-protein interactions (PPIs), the unique biophysical and dynamic properties of Ab-Ag complexes challenge standard PPI models [8]. Recent deep learning approaches for PPIs–such as fingerprint-based methods (MaSIF [9], dMaSIF [10], and PeSTo [11])–offer innovative strategies for modeling these interactions. In particular, dMaSIF represents an advancement over the MaSIF model by directly utilizing atomic coordinates for the analysis of protein surfaces. This approach eliminates the need for precomputed meshes, resulting in a speed improvement of 600 times while maintaining comparable or superior performance levels. The model offers two key functionalities: dMaSIF-site, which predicts interaction sites on protein surfaces, and dMaSIF-search, which identifies potential binding partners through surface matching. Concurrently, antibody-specific tools (EMPM [12], PECAN [13], Surface ID [14]) have been developed to capture the unique structural and energetic features of Ab-Ag binding. Moreover, the Geometric Epitope-Paratope (GEP) method [15] leverages geometric molecular representations to identify binding sites, revealing that while surface-based models excel in epitope prediction, graph-based approaches are particularly effective for paratope prediction–collectively establishing a new state-of-the-art in predicting both epitopes and paratopes.

Capturing antibody flexibility is crucial for enhancing the accuracy of Ab-Ag interaction models [3,16–20] and performing inverse folding [21]. Both backbone and side-chain movements significantly influence binding affinity and specificity, with the paratope displaying considerable conformational variability [18,19]. Increased rigidity can improve binding affinity [22], while enhanced flexibility may allow better tolerance to antigenic sequence variations [23]. This balance is particularly critical when targeting polymorphic antigens or rapidly evolving viral surface proteins, such as those found in HIV-1, coronaviruses, influenza, and hepatitis C [23]. Upon binding (see Fig 1B), both antibodies and antigens undergo subtle conformational adjustments that integrate intrinsic flexibility with induced rearrangements to optimize molecular recognition [15]. Structural analyses reveal minimal backbone movement in non-CDR-H3 loops, with CDR-L2 being notably rigid [24], whereas the CDR-H3 loop shows high variability, facilitating adaptation to diverse epitopes [25]. Similarly, side-chain flexibility, particularly among solvent-exposed aromatic residues like Tyr and Trp, plays a key role in optimizing complementarity at the binding interface [26–28]. Moreover, the formation of Ab-Ag complexes is driven by hydrophobic clusters enriched in aromatic residues, where side-chain dynamics are essential for interface stabilization [29].

Despite significant progress, current deep learning models still struggle with predicting large-scale conformational changes [17]. Although molecular dynamics simulations offer detailed insights into these dynamics, their high computational cost has motivated the exploration of alternative strategies to capture protein flexibility. Recent studies have shown that AlphaFold2’s predicted Local Distance Difference Test (pLDDT) correlates with protein flexibility [30–33]. While earlier works, such as Carugo (2023) [34], emphasized that pLDDT values should not be interpreted as direct indicators of physical flexibility–arguing instead that they reflect only model confidence–this view has been challenged by more recent experimental validations. For instance, Ma et al.[30] employed AF2-generated structures to predict backbone N-H *S*^2^ order parameters from NMR data using a local contact model that incorporates peptide plane interactions, showing that lower pLDDT values correspond to increased flexibility, especially in loop regions. Gavalda-Garcia et al. [33] compared AF2 pLDDT scores with NMR dynamics, MD simulations, and NMA-calculated flexibility, confirming that high pLDDT scores generally indicate stable regions while low scores denote disordered, dynamic regions. However, this metric does not fully capture the entire range of conformational variability. The inherent flexibility of antibody CDRs, particularly CDR-H3, underscores the significance of these findings, as dynamic flexibility is essential for effective antigen recognition and binding. To address this, ITsFlexible [35] was recently introduced as a supervised model for predicting loop flexibility. ITsFlexible classifies CDR3 loops as either rigid or flexible, offering a task-specific and biologically grounded alternative to confidence metrics like pLDDT.

In this study, we systematically examine the impact of flexibility on Ab-Ag interactions. First, we assess the use of pLDDT as a proxy for residue and side-chain flexibility, comparing pLDDT and ITsFlexible predictions. We then extend state-of-the-art fingerprint-based methods to integrate pLDDT and ITsFlexible. We validated the resulting framework by comparing our paratope-epitope predictions with those from GEP. Notably, our approach sets new benchmarks for paratope prediction, underscoring the benefits of incorporating flexibility into antibody modeling. These results indicate that while pLDDT does not directly measure antibody flexibility, it encodes information related to conformational dynamics that can be exploited in the rational design of antibodies with improved breadth or affinity. Finally, we introduce a comprehensive pipeline that combines antibody and antigen sequence inputs with state-of-the-art deep-learning–based pre-folding methods. Our findings reveal how different folding approaches influence overall prediction performance.

## Results

### pLDDT tracks known properties of antibody flexibility

AF2’s pLDDT has been extensively analyzed to understand its relationship with structural flexibility. In our study, we utilize dMaSIF, a fingerprint method that computes molecular features more efficiently than the original MaSIF. To maintain the speed of the method, we needed to derive pLDDT without MSAs while ensuring optimal antibody folding performance. To achieve this, we adopted ESMFold (see Fig 1C), which utilizes ESM-2 embeddings to predict structures without MSAs, surpassing AF2 in performance on single-sequence inputs and providing faster predictions [36,37]. Furthermore, ESMFold offers an optimal balance of accuracy, runtime, and resource consumption, making our model accessible to researchers with limited resources. However, to our knowledge, ESMFold has not been investigated in the context of antibody flexibility. Therefore, we assessed ESMFold predictions for antibody folding, hypothesizing that the CDR loop regions–particularly the highly flexible CDRH3–would display lower pLDDT scores, in alignment with prior research findings [24,25].

**Fig 2:**
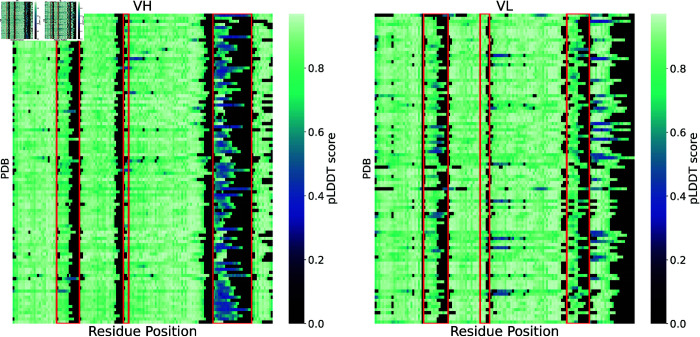
Heatmap representation of pLDDT for VH and VL chains in selected dataset samples. Each row of the two heatmaps represents the same PDB from which the VH and VL are taken, and each column corresponds to the same position numbered with ANARCI. Darker colors indicate lower pLDDT scores (less confident predictions), while lighter colors indicate higher scores (more confident predictions). Each red box represents the CDRs. As shown in Table 1, CDRH3 has lower mean pLDDT values, suggesting higher structural variability or disorder, while CDRL2 has higher mean pLDDT values, indicating higher model confidence and potential rigidity.

Fig 2 illustrates this hypothesis with representative data from our dataset, showing distinctly lower pLDDT values in CDRH3 compared to the higher scores observed in CDRL2, effectively capturing CDR flexibility as detailed in [24,25].

**Table 1:** Mean pLDDT values for antibody regions in VH and VL. Mean pLDDT scores across antibody regions in VH and VL chains. Scores are averaged over all structures in the dataset. Higher pLDDT values indicate greater confidence in the predicted local structure. Framework regions (non-CDRs) consistently exhibit the highest pLDDT values, indicating that they are more structurally stable and confidently predicted. Notably, CDRH3 exhibits the lowest average confidence among all regions, while CDRL2 shows the highest among CDRs.

Region	VH	VL
Whole sequence	0.85 ± 0.04	0.87 ± 0.04
Non-CDRs Regions	0.88 ± 0.02	0.88 ± 0.04
Non-CDR3 Regions	0.87 ± 0.03	0.87 ± 0.04
CDR1	0.82 ± 0.07	0.81 ± 0.09
CDR2	0.80 ± 0.10	0.86 ± 0.07
CDR3	0.66 ± 0.16	0.80 ± 0.07

Table 1 compares pLDDT values for the entire antibody sequence (VH and VL) to those calculated after excluding the CDR regions, particularly CDR3. The analysis also evaluates each region individually. Our findings show that sequences without CDRs have significantly higher mean pLDDT scores than those with CDRs, with differences reaching robust statistical significance (*p*<0.001) in both the VH and VL domains, as determined by a Wilcoxon test. We also conducted a b-factor analysis, which revealed that pLDDT offers a better qualitative approximation of antibody flexibility (see S1 Fig).

Overall, these results suggest that ESMFold-generated pLDDT scores can serve as a *rough proxy* for structural flexibility in antibody models.

#### Zero-shot pLDDT vs. supervised flexibility prediction in CDR3 loops.

ITsFlexible [35] is a recent supervised model that classifies CDR3 loops as rigid or flexible, achieving strong performance on both crystal structures and MD simulations. To benchmark ESMFold’s pLDDT in this context, we replicated the ITsFlexible evaluation protocol using the same test sets. Specifically, we computed the average pLDDT score across each CDR3 loop and applied a threshold of 80%, following the gradation framework proposed by [33], where values above 80% indicate high-confidence rigidity and lower values suggest varying degrees of flexibility. For ITsFlexible, which outputs probabilities, we adopted a threshold of 0.34–values below this indicate confident predictions of rigidity. Using these thresholds, we performed binary classification and assessed both classification metrics (Accuracy, F1 score, Matthews Correlation Coefficient [MCC]) and ranking metrics (AUC-ROC and AUC-PR). As shown in Table 2, ITsFlexible substantially outperforms the zero-shot pLDDT baseline in AUC-PR, particularly on the functionally critical and structurally diverse CDRH3 region (0.82 vs. 0.44). This supports the effectiveness of task-specific supervision in capturing biologically relevant flexibility. Interestingly, despite *not being designed as a flexibility predictor*, pLDDT attains higher Accuracy and F1 on CDRH3, suggesting it encodes some structural signals correlated with flexibility. As also noted in [33], pLDDT is better interpreted as a binary indicator of structural reliability rather than a continuous measure. In line with this, in our models we treat both pLDDT and ITsFlexible as binary features, making classification-oriented metrics (Accuracy, F1) more informative than AUC-ROC and AUC-PR scores. Importantly, the inclusion of a random and majority-class baselines provides additional context. Both ITsFlexible and pLDDT perform above random guessing and trivial majority prediction across most metrics, indicating that they capture informative signals beyond dataset imbalance.

**Table 2:** Comparison between ITsFlexible and ESMFold’s pLDDT scores with random and majority class baselines. Evaluation across classification and ranking metrics shows that while pLDDT achieves higher F1 and Accuracy on CDRH3, ITsFlexible achieves superior AUC-ROC and AUC-PR, highlighting its strength in identifying flexible regions.

Model	Loop	Accuracy	F1	MCC	AUC-ROC	AUC-PR
ITsFlexible	CDRH3	0.49	0.17	**0.21**	**0.76**	**0.82**
	CDRL3	0.47	**0.43**	0.11	**0.68**	**0.54**
pLDDT	CDRH3	**0.58**	**0.72**	0.10	0.29	0.44
	CDRL3	0.69	0.40	**0.19**	0.37	0.22
Majority	CDRH3	0.56	0.71	0.00	0.50	0.56
	CDRL3	**0.73**	0.00	0.00	0.50	0.27
Random	CDRH3	0.51	0.53	0.03	0.52	0.58
	CDRL3	0.51	0.36	0.01	0.50	0.28

These results highlight distinct strengths: pLDDT provides a coarse binary signal that is competitive with supervised models for classification, while ITsFlexible offers superior ranking ability and precision in identifying flexible residues. Given these strengths, we incorporate ITsFlexible predictions into our model as an alternative representation for CDR3 flexibility.

### Flexibility improves antibody-antigen interaction prediction

To address the antibody–antigen interaction challenge, we evaluated the dMaSIF model under three configurations: one without flexibility ("no flex"), one incorporating flexibility as an additional feature ("flex"), and one with iterative flexibility ("iter"), where the flexibility feature is reintroduced in the embeddings during convolution. The baseline model employs two primary feature groups, geometric and chemical, which we augmented by adding a flexibility feature. Specifically, we investigated four approaches to encode flexibility: residue flexibility ("res"), binary residue flexibility ("res bin", assigned a value of 1 if pLDDT>80 according to [33], otherwise 0), atomic flexibility ("atomic") and binary atomic flexibility ("atomic bin"). Moreover, we considered the same model configuration of iterative flexibility, adding the ItsFlexible binary prediction instead of the pLDDT. Finally, we included a baseline using the one-hot encoding of CDRH3. We focused on CDRH3 because Table 1 shows that residues with pLDDT<80% are predominantly located in this loop, which is also the primary determinant of antigen binding. This baseline allows us to assess whether the signal is already captured by CDRH3 alone or whether flexibility provides additional information. More details are provided in Method.

**Fig 3:**
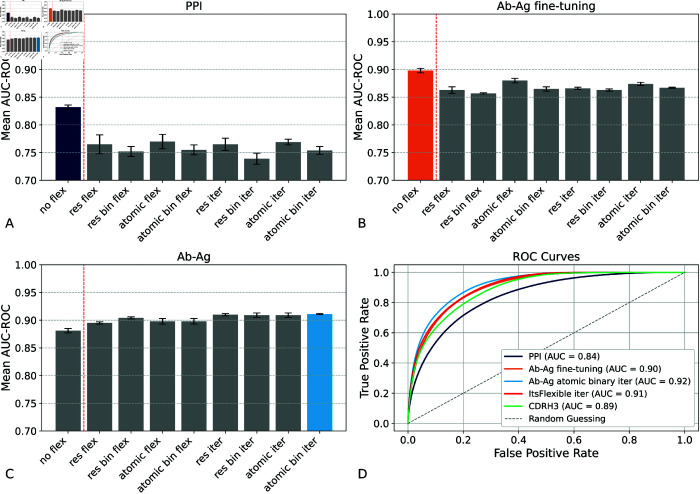
ROC-AUC for the different models. The bar plots present the results of the different models for 5 runs. A) "PPI" represents the model trained on PPI data and applied to Ab-Ag complexes; B) "Ab-Ag fine-tuning" corresponds to the model pre-trained on PPI and subsequently fine-tuned on Ab-Ag complexes; C) "Ab-Ag" denotes the model trained directly on Ab-Ag data. Within each bar plot, "no flex" refers to the baseline model, while "res" (residue flexibility), "atom" (atomic flexibility), and "iter" (iterative flexibility) indicate different modeling approaches incorporating flexibility. The highest-performing model in each category is highlighted in a different color. D) ROC curves of the highest-performing models for each sub-plot, our model with ITsFlexible representation, and the baseline with the one-hot encoding of CDRH3. Overall, iterative flexibility models demonstrate the best performance.

Initially, the model was trained using PPI data (dMaSIF dataset). As shown in Fig 3A, adding flexibility during the PPI training did not improve the predictions for the PPI or Ab-Ag tasks. Subsequently, we fine-tuned the PPI-trained model with Ab-Ag data filtered from SAbDAb[38] (Fig 3B), which yielded overall performance gains; yet, the flexible variant did not outperform its non-flexible counterpart in this fine-tuning stage. Notably, among the various methods explored for incorporating pLDDT-derived flexibility, atomic flexibility consistently produced the most favorable outcomes. We trained the model from scratch on Ab-Ag data and showed that integrating flexibility significantly improved performance, with the binary model at atomic resolution achieving better results for both flexibility and iterative flexibility configurations (Fig 3C). These findings suggest that Ab-Ag interactions constitute a distinct category of PPIs and underscore the necessity of developing a dedicated model that better characterizes these interactions, particularly by leveraging flexibility to improve predictive accuracy. Following the original dMaSIF-search setup, the task was evaluated by comparing interaction surface points to an equal number of non-interacting surface points from both proteins. This yields a balanced dataset where AUC-ROC and AUC-PR provide equivalent information; thus, only AUC-ROC is reported, as done in the original work. Fig 3 illustrates the results of the different models for five runs and the corresponding ROC curves, based on the original study that evaluated the PPI task using AUC-ROC derived from the final embedding score. We performed a paired t-test comparing the ”no flex Ab-Ag fine-tuning” model to our best-performing model, ”atomic bin iter Ab-Ag” (shown in light blue and orange in Fig 3, respectively). The analysis revealed a statistically significant performance improvement, with the former achieving a mean score of 0.892 (95% CI: [0.878, 0.906]) and the latter a mean of 0.916 (95% CI: [0.907, 0.925]), with a p < 0.05.

The results presented in Fig 3 indicate that pre-training on PPI data does not enhance fine-tuning with Ab-Ag complexes. While this may seem counterintuitive, it highlights fundamental differences between PPIs and Ab-Ag interactions, particularly in the manifestation of flexibility within each context. In fact, PPIs typically involve broad, dynamic interfaces influenced by hydrophobic, electrostatic, and van der Waals forces, with conformational changes occurring across extensive surface regions [39]. In contrast, Ab-Ag interactions are generally more specific, often governed by localized flexibility within the antibody’s CDRs, and frequently adhere to “lock-and-key” or “induced fit” binding models [24,25,40]. Due to these differences, a model pre-trained on PPIs may acquire representations of flexibility that do not effectively transfer to Ab-Ag contexts, thereby limiting the advantages of fine-tuning with flexibility features. Further analysis of this concept will be conducted in the subsequent Sect “Chemical and Flexibility Features are the most Relevant for the Final Prediction". Finally, we see that dMaSIF-search, utilizing ITsFlexible predictions for flexibility, achieves interesting performance; however, pLDDT still yields the highest results among the different configurations. The baseline model based on a one-hot encoding of CDRH3 performs worse than the flexibility-based models, underscoring the added value of incorporating flexibility information. Further analysis of the components most relevant for the predictions is provided in S6 Fig for ITsFlexible and in S7 Fig for the CDRH3 one-hot baseline.

#### Binary flexibility patterns emerge despite linear flexibility modeling.

**Fig 4:**
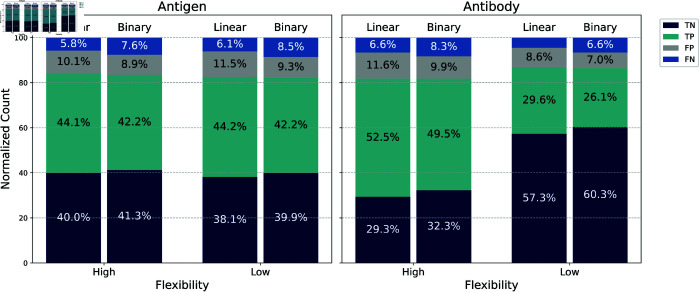
Normalized distribution of predictions and their relationship with flexibility. Each bar plot represents the normalized distribution of true positives (TP), true negatives (TN), false positives (FP), and false negatives (FN) produced by two predictive models: one trained on linear pLDDT scores (Linear) and one trained on binarized pLDDT values (Binary). Results are grouped based on residue flexibility, classified as either high or low using a threshold of 80%. Bars are stacked to show the relative proportion of each outcome category within each flexibility group. Linear and binary model bars exhibit similar behaviors, indicating that the linear model learns a binary representation of flexibility. Notably, both models tend to produce more accurate predictions (higher TP and TN rates) for low-flexibility regions for the antibody context, suggesting that structural stability contributes to improved prediction reliability.

Model results indicate that binary pLDDT thresholds (e.g., using a cutoff at 80) are generally more effective for modeling flexibility. In this section, we examine whether a model trained on continuous (linear) pLDDT values also displays predictive behavior that aligns with binary flexibility representations. Specifically, we compare the predictions from the residue-level linear flexibility model ("Linear")–our second-best performing model–with those from our top-performing model, which uses binary atomic flexibility ("Binary"). To explore this, we classified predictions into four categories–true positives (TP), true negatives (TN), false positives (FP), and false negatives (FN)–and grouped them by high (pLDDT ≤ 80%) and low (pLDDT > 80%) flexibility using a post-hoc binning of the pLDDT values. These distributions are visualized for both antigen and antibody surfaces in Fig 4.

A key observation from the figure is that, for both antigen and antibody, each Linear/Binary pair of bars within the same flexibility class exhibits highly similar proportions of TP, TN, FP, and FN predictions. This consistent similarity suggests that the Linear model, although trained on continuous values, has implicitly learned to treat flexibility in a threshold-like way. In effect, the model appears to have internalized a binarized representation of flexibility during training–demonstrating an emergent binary behavior despite receiving linear input.

Beyond this general similarity, we observe a clear pattern in the antibody predictions:

- In low-flexibility regions, both models produce a higher proportion of true negatives (Linear: 57.3%, Binary: 60.3%), indicating strong correlation between low flexibility and non-interacting surface regions.
- In high-flexibility regions, both models yield a greater proportion of true positives (Linear: 52.5%, Binary: 49.5%), suggesting that flexible regions are more likely to be correctly identified as interaction sites.

In contrast, the antigen model shows no such pattern: the TP and TN rates are balanced across flexibility classes, and the distributions remain relatively constant between the two models. This indicates that flexibility is not a discriminative feature for antigen interaction site prediction in the same way it is for antibodies.

These findings highlight two key conclusions:

1. Even when trained on continuous pLDDT values, the Linear antibody model demonstrates binary-like predictive behavior, supporting the practical value of using binary flexibility representations.
2. Flexibility is a more informative feature for antibody surface modeling than for antigen surfaces, reinforcing its relevance for capturing paratope dynamics in antibody–antigen interactions.

Additional analysis on the pLDDT distribution and the interacting and non-interacting points are presented in S3 Fig for the binary pLDDT model and in S4 Fig for the linear pLDDT model.

#### Chemical and flexibility features are the most relevant for the final prediction.

**Fig 5:**
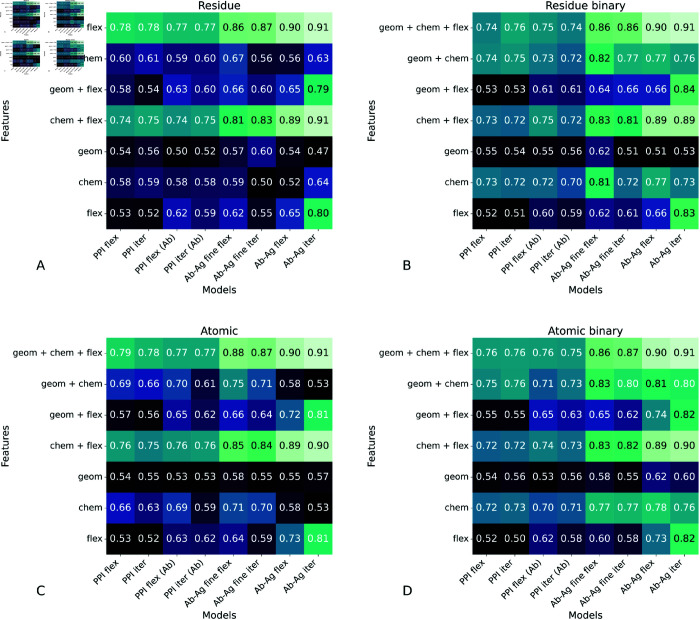
Ablation study for the different combination of features. In the graph, "geom" denotes geometrical features, "chem" represents chemical features, and "flex" indicates flexibility. The models represent those trained on PPI data ("PPI") and applied to Ab-Ag complexes ("PPI (Ab)"); a model fine-tuned on Ab-Ag complexes ("Ab-Ag fine"); and a model trained from scratch ("Ab-Ag"). For each model, two flexibility additions are considered: flexibility ("flex") and iterative flexibility ("iter"). The results are for the A) Residue, B) Residue Binary, C)Atomic, and D) Atomic Binary pLDDT.

**Fig 6:**
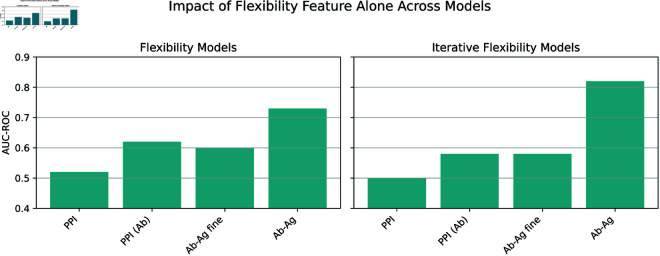
Impact of flexibility features on model performance across training strategies. Bar plots showing the ROC-AUC performance of models using only the flexibility feature (left) and the iterative flexibility variant (right) across four training configurations: models trained on protein–protein interactions (PPI), PPI models applied to antibody–antigen (Ab–Ag) data without fine-tuning (“PPI (Ab)”), PPI models fine-tuned on Ab–Ag data (“Ab–Ag fine”), and models trained from scratch on Ab–Ag data (“Ab–Ag”).

The model results presented in Fig 3 indicate that PPI pre-training does not improve post-fine-tuning performance on Ab-Ag interactions. While we previously elucidated this phenomenon in biological terms, we also aimed to analyze the results from a modeling perspective. To this end, we conducted an extensive ablation study to dissect the contributions of various model features in predicting PPI and Ab-Ag interactions, with a particular focus on the role of flexibility derived from pLDDT. In our investigation, we systematically evaluated all possible combinations of three feature groups: geometrical, chemical, and flexibility. As illustrated in Fig 5A-D, while all methods benefit from integrating chemical and flexibility features, the relative importance of each feature set varies across models. PPI interaction models primarily rely on chemical attributes, whereas the fine-tuning model for antibody-antigen interactions demonstrates an increasing significance of flexibility features. Analogous trends were observed in the Ab-Ag model trained from scratch, thereby reaffirming that pLDDT-based features are robust predictors. These results underscore the critical role of the flexibility score in enhancing Ab-Ag interaction predictions. To provide a clearer overview of these results, Fig 6 illustrates the data presented in Fig 5D regarding the flexibility feature. We have chosen to represent Fig 5D because the atomic and binary representations with iterative flexibility yielded the best model. This figure demonstrates that the impact of flexibility on protein-protein interactions (PPI) is approximately 0.5, with an increase observed for antibody inference (PPI(Ab)), while remaining unchanged following fine-tuning. These findings indicate that the PPI-learned representation is not optimally updated to utilize flexibility within the antibody-antigen domain. For the ablation study on ITsFlexible, refer to S6 Fig.

A paired t-test was conducted on the model trained with atomic binary flexibility features (our best-performing model), comparing the original feature set (geom + chem + flex) to alternative combinations. The analysis revealed that only the chem + flex combination did not differ significantly from the original (p > 0.01), whereas all other combinations showed statistically significant differences (p < 0.01). Additional information is in S2 Table. These results suggest that including chemical and flexibility features is sufficient to maintain performance.

### pLDDT enhances paratope-epitope prediction

**Fig 7:**
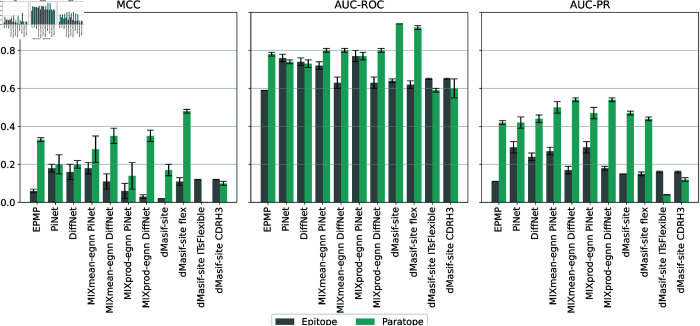
Paratope–epitope prediction. Results of five runs in terms of MCC, AUC-ROC, and AUC-PR. The plot compares different GEP models, dMaSIF-site, dMaSIF-site flex (our model), dMaSIF-site ITsFlex (our model), and dMaSIF-site CDRH3 (one-hot encoding CDRH3). For the GEP models, we report the results from the original GEP publication, as model weights were not publicly available. In contrast, all dMaSIF-variants were trained and evaluated on the same dataset used in the original study.

For paratope and epitope prediction, we fine-tuned our model using the GEP dataset [15], which currently serves as the most comprehensive and up-to-date benchmark for this task. The GEP framework has demonstrated superior performance compared to several state-of-the-art methods, including EPMP [12], DiffNet [41], and PiNet [42]. Following prior work, we evaluated our model using MCC, AUC-ROC, and AUC-PR–three metrics that are particularly suitable for imbalanced binary classification problems such as residue-level binding site prediction. Results across five independent runs are summarized in Fig 7.

Our model, **dMaSIF-site flex**, improves paratope and epitope prediction compared the original framework. For epitopes, performance is comparable to the state-of-the-art MIXmean-egnn DiffNet (hks), while for paratopes it achieves the highest MCC across all models and baselines (original framework and one-hot encoding of CDRH3). When pLDDT is replaced with ITsFlexible predictions for CDR3 (dMaSIF-site ITsFlexible), paratope performance drops substantially whereas epitope performance remains largely unaffected, supporting the use of pLDDT as an informative signal for antibodies. Although dMaSIF-site flex has slightly lower ROC-AUC and PR-AUC than some models, we emphasize MCC because it better reflects performance under class imbalance. Moreover, most false positives/negatives are within 3 Å of the true interface (S2 Fig), indicating the model consistently identifies the correct region–an aspect better captured by MCC than PR-AUC, which penalizes all errors equally regardless of spatial proximity. Finally, contrary to the original GEP claim that surfaces suit antigens while internal representations suit antibodies, we find that an atomic-level surface representation is also highly effective for antibodies.

**Fig 8:**
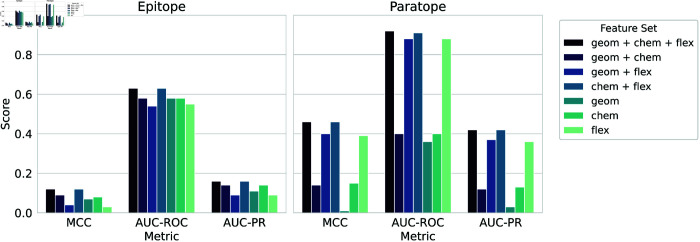
Ablation study for paratope and epitope prediction. The bar plot shows how the MCC, AUC-ROC and AUC-PR change with the different combinations of geometric ("geom"), chemical ("chem"), and flexibility ("flex") features for dMasif-site flex.

To assess feature importance, we conducted an ablation study (Fig 8). The best results for both tasks come from combining chemical and flexibility features. Chemical properties are most informative for epitope mapping, while flexibility is crucial for paratope identification, highlighting the value of dynamic information. Additional ablations for dMaSIF-site ITsFlexible and the CDRH3 baseline S6 and S7 Figs. It is important to clarify that these results do not contradict the previously reported performance of dMaSIF-site, which achieved higher AUC-PR scores than our model. The observed emphasis on different features is a direct consequence of the models’ distinct training inputs. The original dMaSIF-site was trained exclusively on geometric and chemical features, naturally optimizing their use. Our model, by contrast, was trained on an expanded feature set including flexibility, and it adapted by leveraging the strong discriminative power of this additional dynamic information, particularly for predicting paratopes. Our findings are consistent with the original dMaSIF study, which showed that chemical features are more informative than geometric ones. In our case, flexibility appears to complement and even enhance the chemical representation more effectively than geometric features.

Given the importance of flexibility, we further investigated its relationship with classification outcomes. Chi-Square tests for both paratope and epitope classifications rejected the null hypothesis of independence; however, the association was modest, with Cramér’s V values of 0.04 for epitopes and 0.24 for paratopes. Additional analysis of interacting and non-interacting sites in relation to pLDDT values is provided in S5 Fig. These results support our conclusion that pLDDT is an informative feature, but not sufficient on its own to explain paratope classification. The model must still learn to combine pLDDT with chemical and geometric context to make accurate predictions.

### Fold quality affects predictive performance

In our study, the input requirement is an Ab-Ag complex; however, obtaining experimental structures is frequently impractical due to resource limitations. Given that antibody and antigen sequences are more readily available, we incorporated a pre-folding step to facilitate sequence-based inputs. In this approach, ESMFold is used to calculate pLDDT scores per chain, leveraging its superior speed for single-chain predictions, although it is not specifically optimized for complex folding. Recently, three new state-of-the-art methods for complex folding were published: AF3[7], Chai-1[43], and Boltz-1[44]. Chai-1 is a multimodal foundation model for molecular structure prediction that has demonstrated performance comparable to AF3 for protein folding and superior outcomes in antibody folding when compared to AlphaFold Multimer [45], even in the absence of MSAs. Boltz-1 is the first fully commercially available open-source model that achieves AF3-level accuracy in predicting the 3D structure of biomolecular complexes, providing a powerful tool for accurately modeling complex biomolecular interactions. In this section, we evaluate the performance differences when these methods are applied as pre-folding steps, comparing the original PDB structures with the predicted ones. For AF3, we used the server implementation, while for Chai-1 and Boltz-1, we used the source code. Previous studies indicate that AF3, Chai-1, and Boltz-1 exhibit comparable performance when both MSAs and templates are used. Hence, we selected AF3 as our MSA/template-based benchmark and evaluated Chai-1 and Boltz-1 in the absence of both. This worst-case scenario–depriving the models of both evolutionary (MSA) and structural (template) information–establishes a lower bound on their performance, providing insight into the capabilities of our model under adverse conditions. These analyses were conducted on the GEP test dataset, dictated by the AF3 submission constraints.

#### Folding quality declines across models without MSAs or templates.

We predicted the structures of 49 experimentally determined Ab–Ag complexes using AF3, and Chai-1 and Boltz-1 (both run without MSAs or templates: no-MSA/templ.). To quantify the accuracy of the predicted structures, we computed the root-mean-square deviation (RMSD) between the predicted and experimental structures (see Table 3). Chai-1 demonstrated robust performance in predicting antibody structures and the overall Ab-Ag complex. AF3, by incorporating both MSAs and templates, excelled in predicting the individual components, particularly enhancing antigen predictions and yielding lower overall errors in the final complex relative to the other methods. Boltz-1 faced significant challenges in modeling the antigen without MSA and templates, as indicated by the highest RMSD.

**Table 3:** Folding quality for original complexes. Performance of AF3, Chai-1 and Boltz-1 in terms of RMSD between the original complex and the folded version. (no-MSA/templ.) indicates that the model does not use templates and MSA.

Model	RMSD Ab	RMSD Ag	RMSD Complex
AF3	2.88±3.00 Å&2.28±3.30 Å&11.86±7.72 Å
Chai-1 (no-MSA/templ.)	2.79±2.55 Å&7.76±6.98 Å&15.13±5.76 Å
Boltz-1 (no-MSA/templ.)	2.52±2.13 Å&12.41±7.51 Å&16.70±5.52 Å

We subsequently analyzed the confidence metrics derived from the folding predictions to evaluate the quality of the antibody–antigen complexes from both local and global perspectives. The predicted alignment error (PAE) and its inter-chain variant (iPAE) assess the anticipated residue-residue alignment discrepancies, whereas the predicted TM scores (pTM and ipTM) serve as proxies for overall and interface-specific structural accuracy, respectively. The data presented in Table 4 reveal that AF3 consistently outperforms its no-MSA/templ. counterparts when both global and interface-specific metrics are considered. AF3 achieves the lowest average PAE and iPAE, indicating tighter global packing and more precise Ab-Ag contacts than Chai-1 or Boltz-1 (no-MSA/templ.). In contrast, Chai-1 slightly edges out AF3 in terms of global topology as reflected by a marginally higher pTM score; however, there is a slight loss in performance for the ipTM, underscoring AF3’s superior ability to recapitulate the precise interfacial geometry essential for antigen recognition. Among the evaluated models, Boltz-1 input exhibited inferior performance compared to Chai-1, as evidenced by lower scores in both global (pTM) and interface-specific (ipTM) metrics. Moreover, the ipTM can be considered an indicator of docking quality, demonstrating a linear correlation between DockQ and ipTM for the heavy chain and antigen [46].

**Table 4:** Folding quality. Performance of AF3, Chai-1 and Boltz-1 (no-MSA/templ.) in terms of PAE, iPAE, pTM, and ipTM. (no-MSA/templ.) indicates that the model does not used templates and MSA.

Model	PAE	iPAE	pTM	ipTM
AF3	13.81±3.90	16.43±4.25	0.66±0.12	0.59±0.17
Chai-1 (no-MSA/templ.)	13.94±3.28	20.35±3.92	0.68±0.08	0.55±0.09
Boltz-1 (no MSA/templ.)	16.28±3.43	23.91±4.14	0.63±0.09	0.52±0.10

Considering both the RMSD and the predicted confidence scores, AF3 demonstrated the best overall performance. This result highlights the essential role of MSA and templates in accurate antigen modeling and maintaining a high-quality binding interface. To quantitatively assess interface accuracy, we recommend evaluating the iPAE and ipTM.

#### AF3 and Chai-1 (no-MSA/template) effectively preserve prediction quality.

Based on these findings, we further compared the performance of our model on GEP test set for the Ab-Ag interaction task. Table 5 reports the performance in terms of AUC-ROC for the original and the folded complexes. The folded complexes result in a reduction in model performance, although the decrement is minimal, particularly for AF3. These results suggest that these folding methods can be applied as a pre-processing step with only a modest impact on the overall performance.

**Table 5:** AUC-ROC values for different models and conditions. Comparison of the complex folded with AF3, Chai-1, and Boltz-1 against the performance of the crystal structure for the Ab-Ag interaction task. The results are shown in terms of AUC-ROC.

Category	AF3	Chai-1 (no MSA)	Boltz-1 (no MSA)
Original	0.89 ± 0.05		
Folded	0.88 ± 0.05	0.86 ± 0.07	0.85 ± 0.05

**Fig 9:**
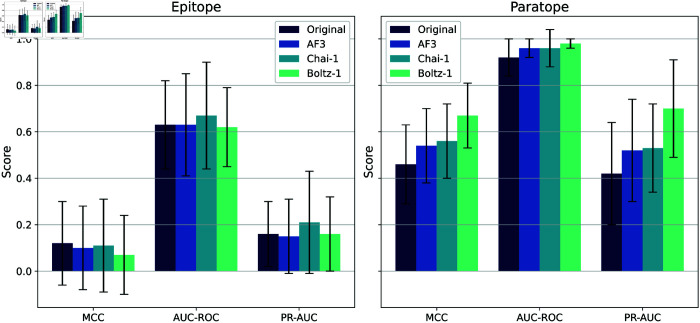
Impact of AF3, Chai-1 and Boltz-1 (no-MSA/templ.) on epitope and paratope prediction. Comparison of AF3 and Chai-1 in terms of MCC, AUC-ROC, and AUC-PR for paratope and epitope prediction. These results are from one model run.

Fig 9 illustrates the performance of paratope and epitope prediction. AF3 closely approximates the performance of the original complex, confirming that models with MSAs and templates most effectively preserve the native structural features. In contrast, Chai-1 shows a modest performance change, attributed to a higher degree of complex modification compared to AF3 (see Table 3). Boltz-1 demonstrates a lower performance in epitope prediction and significantly alters paratope prediction, likely due to having the highest RMSD among the three models.

Interestingly, all three structure-predicted models–Boltz-1, Chai-1, and AF3–outperform the crystal structure in at least one prediction task. This may be attributed to the fact that these models predict the structure of individual components and the docked complex simultaneously. In doing so, they can introduce interface adjustments that enhance surface complementarity. For instance, Pereira et al. [47] found that AlphaFold-Multimer and AlphaFold3 perform poorly on protein-protein heterodimers with small interfaces. Similarly, Hitawala et al. [46] report that AF3 frequently predicts incorrect antigen interfaces, with docking success increasing from 13% to 60% only when sampling 1,000 stochastic seeds–a result that is not reproducible under standard inference settings. More recently, Boltz-2 [48] demonstrated improved docking performance over Boltz-1 in the antibody–antigen setting, yet still falls short of AF3 in predictive accuracy. These findings collectively highlight both the potential and current limitations of generative structure prediction models in modeling antibody–antigen complexes.

Overall, these findings indicate that the use of MSAs and templates may offer a slight advantage in preserving interface quality. Chai-1 (no-MSA/templ.) offers a viable alternative for users unable to access AF3 (e.g., due to data leakage) or use MSA or templates (e.g., limited resources). For a more comprehensive analysis of the performance change about RMSD, refer to S8 Fig.

## Discussion

Antibody–antigen interactions are fundamental to immune recognition and pathogen neutralization. Although numerous computational models have attempted to simulate these interactions, many do not adfequately consider the inherent flexibility of antibodies. While molecular dynamics simulations offer detailed dynamic insights, their high computational cost has prompted the exploration of alternative strategies. Similarly, although b-factors derived from X-ray crystallography provide a measure of flexibility, they are frequently affected by artifacts associated with crystal packing and refinement [49]. Recent research has established a clear link between pLDDT scores and protein flexibility.

By using atomic pLDDT scores as an approximate indicator for structural flexibility, our approach significantly enhances predictive accuracy, achieving a 4% improvement and an AUC-ROC of 92%. Notably, the model variant incorporating flexibility outperformed the other models where flexibility is less relevant, highlighting the importance of dynamic conformational modeling. Furthermore, our method achieved state-of-the-art performance in paratope prediction, confirming that flexibility is a critical factor in the final prediction. Finally, we also explored the impact of various folding methods on model predictions across tasks, revealing the potential to extend our approach to sequence data.

One current limitation of our approach is its dependence on Ab–Ag complex structures, which requires a pre-folding step when using sequence-based data. This introduces computational overhead and limits real-time applicability. To address this, future work will explore structural distillation methods that map fingerprint-level representations directly onto sequences, thereby reducing computational demands and improving practical usability in settings where only antibody and antigen sequences are available. Additionally, the representation of flexibility remains an open research question. While prior studies have investigated correlations between pLDDT and conformational variability, more recent efforts have introduced dedicated tools to quantify residue-level flexibility in proteins [50], offering a promising direction for improved modeling. In parallel, we aim to further explore the potential of the ITsFlexible representation, which offers a targeted classification of CDR3 loop flexibility. Although our ablation studies suggest that loop-level flexibility alone is less informative than atomic-level features for paratope and epitope prediction using surface models, we believe ITsFlexible may hold greater value in other contexts. In particular, we plan to investigate its integration into graph-based models and sequence-based architectures, where its lightweight and interpretable nature could serve as a practical and biologically meaningful signal for flexibility.

This line of investigation aligns with broader immunological insights linking flexibility to antibody function. Notably, increased antibody rigidity has been associated with enhanced antigen-binding affinity [22], while greater conformational flexibility can provide improved tolerance to sequence variations in target antigens [23]. This balance is particularly critical when addressing polymorphic antigens or surface proteins of rapidly evolving viruses such as HIV-1, coronaviruses, influenza, and hepatitis C [23]. Our study demonstrates that pLDDT can effectively model antibody flexibility. Therefore, future advancements in model design should integrate antibody flexibility to develop novel CDRs, computing a trade-off between affinity and breadth based on the objective of the design and the target of interest.

## Materials and methods

In this section, we provide an in-depth overview of the dataset alongside the state-of-the-art methodological framework employed in our investigation, which extends the work described in [51]. Our approach is further enhanced by incorporating innovative analytical techniques focused on pLDDT evaluation, supplemented by rigorous analyses of atomic and binary flexibility. Additionally, we introduce a preliminary validation of ESMFold data, setting the stage for a series of novel experiments and the exploration of emerging tasks, including paratope and epitope predictions.

### Dataset

In this study, we adopt a dual-faceted training approach by harnessing both PPI data and Ab-Ag complexes to rigorously assess our method. For the PPI task, we used the dMaSIF dataset [10], which comprises 4,943 protein-protein complexes. This dataset is systematically partitioned into training and validation sets–with 10% reserved for validation–and complemented by an independent test set of 959 complexes.

For the Ab-Ag complexes, we curated data from the SAbDab database [38] (comprising 16,269 complexes as of April 2024). We meticulously pruned this dataset by eliminating redundant sequences, nanobodies, and complexes with uncharacterized antigens. Subsequent filtering based on structural resolution (below 4 Å) and structural similarity (TM-Score [52] < 30%). We then split the dataset according to the original work of dMaSIF, randomly splitting the data, resulting in 3,032 complexes for training and validation (with 10% held for validation) and a separate test set of 535 complexes (see Table 6).

**Table 6:** Dataset composition. The table presents the composition of datasets used for the different tasks we performed with our model.

Dataset	Training	Validation	Test
PPI	4,449	494	959
Ab-Ag	2,729	303	535
paratope-epitope classification	186	25	49

Furthermore, the embeddings generated by our model serve as the foundation for binary predictions of the paratope and epitope. For this specific task, we employ the benchmark dataset introduced in [15]. The training set is derived from complexes reported in [53], where antibodies exhibit greater than 99% sequence identity while the corresponding antigens maintain sequence identity at or below 90%. Additionally, the validation set, obtained from Docking Benchmark v5 [54], comprises 25 Ab-Ag complexes that share 91% sequence identity with the training data, computed for the entire Ab-Ag complex. The test set includes 49 complexes from SAbDab, ensuring a sequence identity of 70% relative to the training set, also calculated for the full Ab-Ag complex.

### Method

Understanding molecular interactions requires an accurate and detailed representation of protein surfaces, as these features are pivotal in governing PPIs [15]. Our approach extends the dMaSIF framework [10], a sophisticated geometric deep learning tool tailored for surface-based protein analysis. In our method, surface points and their associated normals are meticulously sampled from the protein structure, enabling the computation of mean and Gaussian curvatures across multiple scales. Simultaneously, chemical features–based on atom types and their inverse distances to the sampled points–are extracted and subsequently processed through a multi-layer perceptron (MLP). Protein flexibility is quantified using ESMFold, and this descriptor is integrated with the geometric and chemical information to generate a comprehensive 16-dimensional feature vector. An additional MLP is employed to predict orientation scores for each surface point, which facilitates the alignment of local coordinate systems. This refined feature representation is further enhanced through trainable convolutions and supplementary MLPs, culminating in interaction predictions computed as dot products between the feature vectors of interacting proteins. Finally, for the task of paratope-epitope prediction, we fine-tuned an MLP using the GEP dataset.

#### Data representation.

Proteins are represented as 3D point clouds, capturing the spatial positions of all constituent atoms. Each atom is described using 10 geometric features (5+5 representing mean and Gaussian curvatures) and 6 chemical features (a one-hot encoding for the six most common atom types: C, H, O, N, S, and Se) and one feature of flexibility [0 – 100] predicted by ESMFold. By integrating the pLDDT score, our approach expands this to 17 features.

#### Flexibility score with ESMFold.

ESMFold [37], developed within the ESM2 framework, generates a detailed embedded representation of protein sequences [3]. One of its primary outputs is the pLDDT score, which is computed at the final stage of the folding process. To obtain this score, we processed the relevant sequences using ESMFold, generating a PDB file where the pLDDT values are stored as b-factors for each atom.

**Fig 10:**
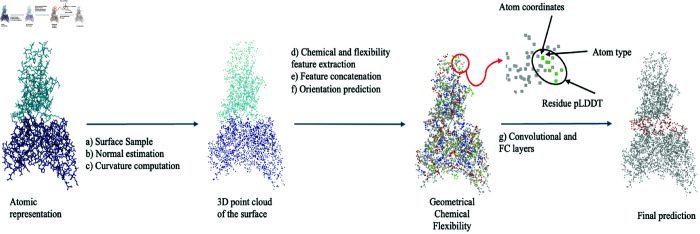
Overview of the method. The protein structure is initially transformed into a 3D point cloud at the atomic resolution, with each atom being characterized by 17 distinct features. A red circle is employed as a visual focal point to illustrate the precise assignment of these features to individual atoms, particularly highlighting residue flexibility as demonstrated in the PDB entry 3ETB. Subsequently, the protein surface is delineated, and the features of its neighboring atoms are incorporated to capture the local environmental context. Finally, our model leverages these detailed descriptors to predict the features of antibody-antigen interactions, ultimately enabling a binary classification of the interaction outcomes.

To account for potential chain flexibility, we explored two types of flexibility: (i) side-chain flexibility, assessed using atomic pLDDT, and (ii) side-chain + backbone flexibility, evaluated using residue-level pLDDT. Furthermore, recent studies indicate that AF2’s pLDDT score may be more effective as a binary classifier for distinguishing ordered and disordered regions rather than as a continuous flexibility metric [33]. Meanwhile, ITsFlexible is a framework that classifies CDR3 loops as flexible or rigid, serving as a specific model for antibodies compared to ESMFold. Based on these insights, we tested five distinct approaches:

- *Residue pLDDT*: We computed the mean pLDDT per residue, assigning a single flexibility score to each atom based on residue-level flexibility, as shown in Fig 10.
- *Residue Binary pLDDT*: Similar to the Residue pLDDT, but with a binary classification; residues with pLDDT > 80 are assigned a score of 100 (low flexibility), 0 otherwise (high flexibility). The threshold of 80 was selected from the work of [33].
- *Atomic pLDDT*: Each atom was assigned its respective pLDDT value, except for hydrogen atoms. Since ESMFold does not compute pLDDT values for hydrogens, we attributed to each hydrogen the pLDDT of its nearest heavy atom (e.g., in an N-H bond, the hydrogen adopts the nitrogen’s pLDDT). This approach is supported by the observation that a heavy atom can influence the NMR signals of adjacent hydrogens (SO-HALA effect) [55].
- *Atomic Binary pLDDT*: Similarly to atomic pLDDT, but with a binary classification; atoms with pLDDT > 80 are assigned a score of 100, 0 otherwise.
- *ITsFlexible*: We used the prediction scores from ITsFlexible to annotate the CDR3 regions. In this configuration, predictions were binarized using a threshold of 0.34, which the original paper defines as indicative of high-confidence rigidity, analogous to 80% threshold for pLDDT, where values above 80% are interpreted as confident rigid regions. However, since our network samples features over the molecular surface, averaging values over the 16 nearest neighbors, binarizing flexibility scores at the surface level would risk diminishing informative variability. To preserve this signal, we retained a linear representation of flexibility on the surface instead of applying a binary cutoff.

For the model using one-hot encoding of the CDRH3, we employed ANARCI (Chothia) to number the CDRH3, establishing a baseline to determine if this information was sufficient compared to the flexibility scores.

#### Model architecture.

**Fig 11:**
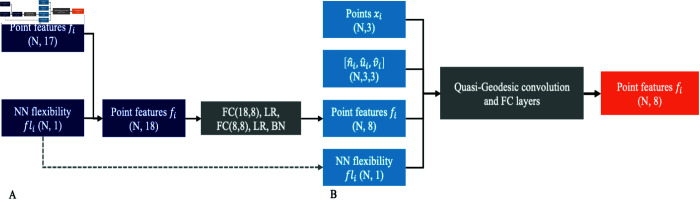
Architecture of iterative flexibility. (A) Initially, the inherent flexibility is seamlessly integrated into the point features, expanding the feature set to 18 distinct attributes. These attributes are subsequently distilled into an 8-element feature vector for each point. (B) In the subsequent stage, a composite input is constructed for the quasi-geodesic convolutions and fully connected (FC) layers. This input comprises the flexibility vector (*fl*_*i*_), the derived 8-element feature vector (*f*_*i*_), the spatial coordinates of the points points(*x*_*i*_), and the descriptors representing local orientation and curvature ($\left[{\hat{n}}_{i},{\hat{u}}_{i},{\hat{v}}_{i}\right]$). Notably, this segment of the network is implemented as a singular layer, which can be iteratively replicated to form multiple layers, ultimately yielding the final output embeddings.

The original architecture employs trainable convolutions, fully connected layers, and batch normalization to refine feature representations. Given that flexibility constitutes only 1/17 of the input features, we introduced iterative flexibility layers to amplify its impact. This modification increased the feature vector size to 18 elements (Fig 11).

In both models, the original and the iterative versions, the interaction predictions were performed using dot product computations between the feature vectors of two proteins, generating interaction scores. These refinements enable our model to capture flexibility-dependent variations in binding interfaces. For the paratope and epitope binary prediction, we added a final MLP. The hyperparameters and training details can be found in S1 Table and S1 Text. 5-fold cross-validation was conducted to select the parameters.

## Supporting information

S1 Table Development environments and requirements. The hardware and software resources used for model development and experimentation.
(XLSX)

S1 Text Model training and hyperparameters. (DOCX)

S2 Table T-test for the combination of different features. T-test comparison between “geom + chem + flex” and other feature sets for the best model. The “chem + flex” features are the only feature combination that is not statistically different. The confidence intervals are set at 95%.
(XLSX)

S1 Fig Comparison between pLDDT and b-factor. We examined the b-factor values, which were normalized as z-scores [56], to serve as an alternative measure of structural flexibility. Interestingly, unlike the pLDDT outcomes, the b-factor analysis indicates that non-CDR regions display higher values relative to the entire sequence, a finding that could suggest greater flexibility. Notably, CDR2 and CDR3 regions systematically exhibit lower b-factors, which may appear counterintuitive given the experimental studies [24,25]. However, this discrepancy is likely influenced by potential inaccuracies inherent in structure determination and confounding non-dynamic factors such as crystal packing [49,57]. As a consequence, b-factors do not reliably serve as proxies for flexibility.
(TIFF)

S2 Fig Location of FP and FN compared to the interface. We evaluated the false positive (FP) and false negative (FN) distributions relative to the interaction boundary for the paratope and the epitope, considering a concave hull and a possible threshold of 3 Å. For epitope predictions, the model tends to generate false positives predominantly outside the hull, indicating that many non-interacting residues are mistakenly classified as part of the epitope. In contrast, most false negatives occur inside the hull–suggesting that essential interacting residues are being missed. In contrast, paratope predictions display a more balanced error distribution; false positives are common both near the hull boundary (within 3 Å ) and outside it, while false negatives are split between those occurring near the boundary and those inside. To quantify concave hull overlap between original and predicted, we calculated the Mean Surface Distance (MSD) and Root Mean Squared Distance (RMSD) by comparing predicted hulls with ground truth for both antigen and antibody structures. The antigen model yielded an MSD of 11.53 ± 11.03 and an RMSD of 16.01 ± 9.09, showing high variability. In contrast, the antibody model achieved a lower MSD of 3.53 ± 7.49, suggesting a closer approximation to the true hull, though its RMSD remains relatively high at 9.28 ± 4.95, likely due to a few extreme mispredictions. These results highlight both the strengths and limitations of our concave hull approach in capturing Ab-Ag interaction boundaries.
(TIFF)

S3 Fig Relationship between binary pLDDT and interaction sites in antibody–antigen interfaces. **Top row**: Normalized histograms of binary pLDDT values (0 = low confidence/flexible, 100 = high confidence/rigid) for ground-truth interacting (dark blue) and non-interacting (teal) residues. Antibody residues (left) show a higher density of interacting positions in low-pLDDT regions, consistent with expected flexibility of paratopes. In contrast, antigen residues (right) exhibit similar pLDDT distributions across both interacting and non-interacting sites. **Bottom row**: Analogous distributions based on predicted interactions from dMaSIF-search flex, with interacting (blue) and non-interacting (gray) categories. Predicted paratope residues also show greater enrichment in low-pLDDT regions, mirroring the ground truth trend. These results support the hypothesis that structural flexibility, as captured by low pLDDT, correlates with interaction propensity primarily on the antibody side.
(TIFF)

S4 Fig Relationship between linear pLDDT and interaction sites in antibody–antigen interfaces. **Top row**: Density estimates of the pLDDT distributions for interacting (dark blue) and non-interacting (teal) points in antibodies (left) and antigens (right), based on ground truth labels. Antibody interaction sites show a moderate enrichment in low-pLDDT regions, consistent with increased flexibility at binding interfaces. In contrast, the pLDDT distributions of interacting and non-interacting antigen points are nearly indistinguishable. **Bottom row**: Hexbin scatter plots showing the relationship between predicted interaction probabilities (from dMaSIF-search flex) and pLDDT values for antibody (left) and antigen (right) points. Most high-pLDDT residues have low predicted interaction probabilities, suggesting that rigid regions are generally predicted as non-interacting. The pattern is more pronounced for antibodies, in line with the known flexibility of paratopes. Color bars indicate hexbin counts.
(TIFF)

S5 Fig Normalized histograms of pLDDT for interacting and non-interacting residues in antibody–antigen interfaces, separated by ground truth and predicted contacts. **Top row**: True interaction labels derived from antibody–antigen complex structures are used to separate residues into interacting (dark blue) and non-interacting (teal) classes. Distributions are shown for paratope (left) and epitope (right) residues. Interacting paratope residues are enriched at low-confidence (low pLDDT) regions, suggesting increased local flexibility near true binding sites. **Bottom row**: Predicted interactions from dMaSIF-site flex are used in place of true labels. Predicted interacting residues (blue) and predicted non-interacting residues (gray) show similar trends to the ground truth, with low-pLDDT enrichment in predicted paratopes and more uniform distributions in epitopes.
(TIFF)

S6 Fig Ablation study for paratope and epitope prediction using dMaSIF-site ITsFlexible. The bar plot illustrates how performance metrics (MCC, AUC-ROC, and AUC-PR) vary across different combinations of geometric ("geom"), chemical ("chem"), and flexibility ("flex") features. For Ab-Ag interaction predictions, flexibility and chemical + flexibility features are the most effective, highlighting the importance of using ITsFlexible representation in the model. In the case of epitope prediction, removing the flexibility feature has little impact, highlighting the stronger influence of chemical features. In contrast, for paratope prediction, flexibility, especially when combined with chemical information, proves to be the most impactful. Flexibility alone also shows a strong effect, suggesting that the observed drop in performance compared to pLDDT-based models can be largely attributed to the loss of this specific feature.
(TIFF)

S7 Fig Ablation study of one-hot encodings for CDRH3. With CDRH3-only encodings, in the Ab–Ag interaction task the chemical features contribute most, and performance improves further when they are combined with geometric features. For paratope prediction, the CDRH3 one-hot channel becomes more informative, particularly in combination with chemical features.
(TIFF)

S8 Fig Impact of backbone deviation on predictive performance for antibody-antigen interfaces. Each panel shows the change in prediction quality (*Δ* = experimental – refolded) versus the RMSD between predicted and crystal structures, across three refolding methods (Chai-1, Boltz-1, AF3). *Left*: *Δ* ROC AUC for the full complex shows a slight negative trend, suggesting that higher RMSD leads to marginally lower predictive quality. *Middle*: *Δ*MCC for the antibody interface shows a positive trend, indicating that higher complex RMSD improves paratope prediction because the interface is "compromised". *Right*: *Δ*MCC for the antigen interface mirrors the first panel, exhibiting a weak negative trend as RMSD increases, which reduces performance for epitope prediction.
(TIFF)
