# Supplementary material for: Enhancing antibody-antigen interaction prediction with atomic flexibility
Source: PLoS Comput Biol. 2025 Oct 13;21(10):e1013576. doi: 10.1371/journal.pcbi.1013576 (PMC12530544; doi:10.1371/journal.pcbi.1013576)
Supplement: S1 Text — (DOCX) [file pcbi.1013576.s002.docx]

**Model Training and Hyperparameters**

For pre-training dMaSIF on protein-protein interactions (PPI) and fine-tuning it for antibody-antigen (Ab-Ag) interactions, we used a radius of 9.0, an embedding dimension of 8, and a single layer. When training the Ab-Ag model from scratch, the parameters for the non-flexible version were consistent with those of the original dMaSIF model. For the flexibility-enhanced models, which included configurations both with and without iterative layers, we set the radius to 10.0 and the embedding dimension to 16. Specifically, the model without iterative layers included 3 layers, while the model with iterative layers had 5 layers. All models were trained with a batch size of 8 for 200 epochs, using early stopping, binary cross-entropy loss, and AMSGrad as the optimizer with a learning rate of 3e-4. For the MLP we used AMSGrad with weighted binary cross-entropy loss and a learning rate of 3e-4 for the epitope and 1e-3 for the paratope and 50 epochs. The same configurations were also maintained for the baseline with one-hot encoding of CDRH3.
