# Supplementary figures and images for: Enhancing antibody-antigen interaction prediction with atomic flexibility
Source: PLoS Comput Biol. 2025 Oct 13;21(10):e1013576. doi: 10.1371/journal.pcbi.1013576 (PMC12530544; doi:10.1371/journal.pcbi.1013576)

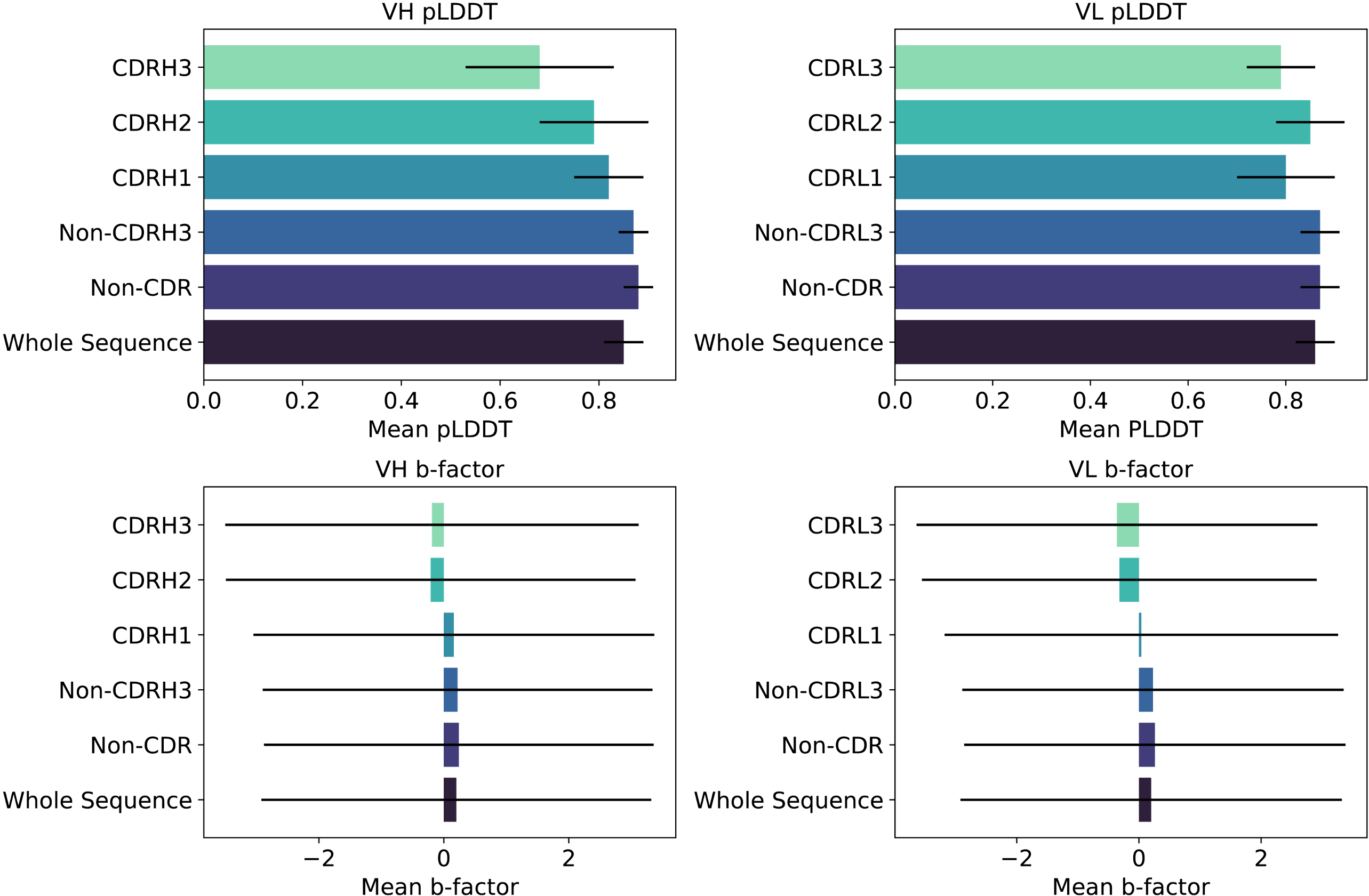

Supplement: S1 Fig — We examined the b-factor values, which were normalized as z-scores [56], to serve as an alternative measure of structural flexibility. Interestingly, unlike the pLDDT outcomes, the b-factor analysis indicates that non-CDR regions display higher values relative to the entire sequence, a finding that could suggest greater flexibility. Notably, CDR2 and CDR3 regions systematically exhibit lower b-factors, which may appear counterintuitive given the experimental studies [24,25]. However, this discrepancy is likely influenced by potential inaccuracies inherent in structure determination and confounding non-dynamic factors such as crystal packing [49,57]. As a consequence, b-factors do not reliably serve as proxies for flexibility. (TIFF) [file pcbi.1013576.s004.tif]

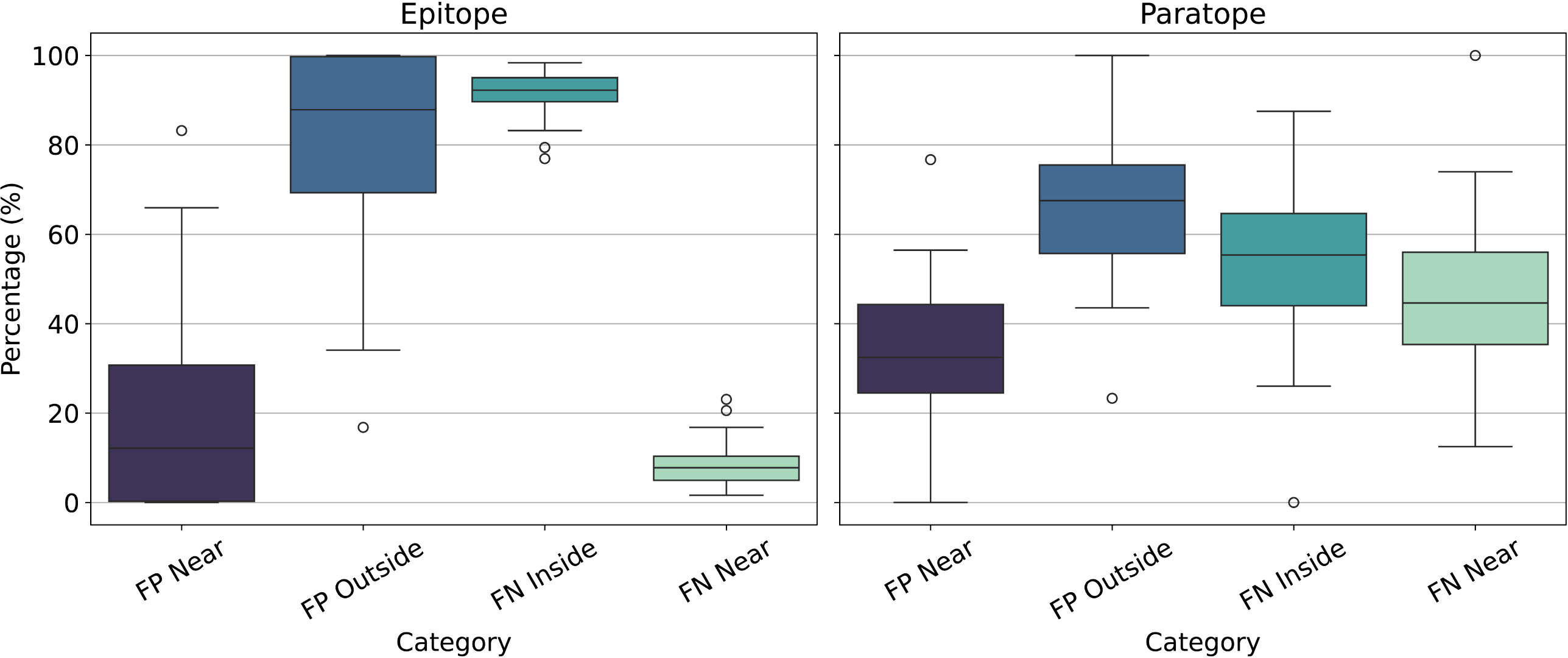

Supplement: S2 Fig — We evaluated the false positive (FP) and false negative (FN) distributions relative to the interaction boundary for the paratope and the epitope, considering a concave hull and a possible threshold of 3 Å. For epitope predictions, the model tends to generate false positives predominantly outside the hull, indicating that many non-interacting residues are mistakenly classified as part of the epitope. In contrast, most false negatives occur inside the hull–suggesting that essential interacting residues are being missed. In contrast, paratope predictions display a more balanced error distribution; false positives are common both near the hull boundary (within 3 Å ) and outside it, while false negatives are split between those occurring near the boundary and those inside. To quantify concave hull overlap between original and predicted, we calculated the Mean Surface Distance (MSD) and Root Mean Squared Distance (RMSD) by comparing predicted hulls with ground truth for both antigen and antibody structures. The antigen model yielded an MSD of 11.53 ± 11.03 and an RMSD of 16.01 ± 9.09, showing high variability. In contrast, the antibody model achieved a lower MSD of 3.53 ± 7.49, suggesting a closer approximation to the true hull, though its RMSD remains relatively high at 9.28 ± 4.95, likely due to a few extreme mispredictions. These results highlight both the strengths and limitations of our concave hull approach in capturing Ab-Ag interaction boundaries. (TIFF) [file pcbi.1013576.s005.tif]

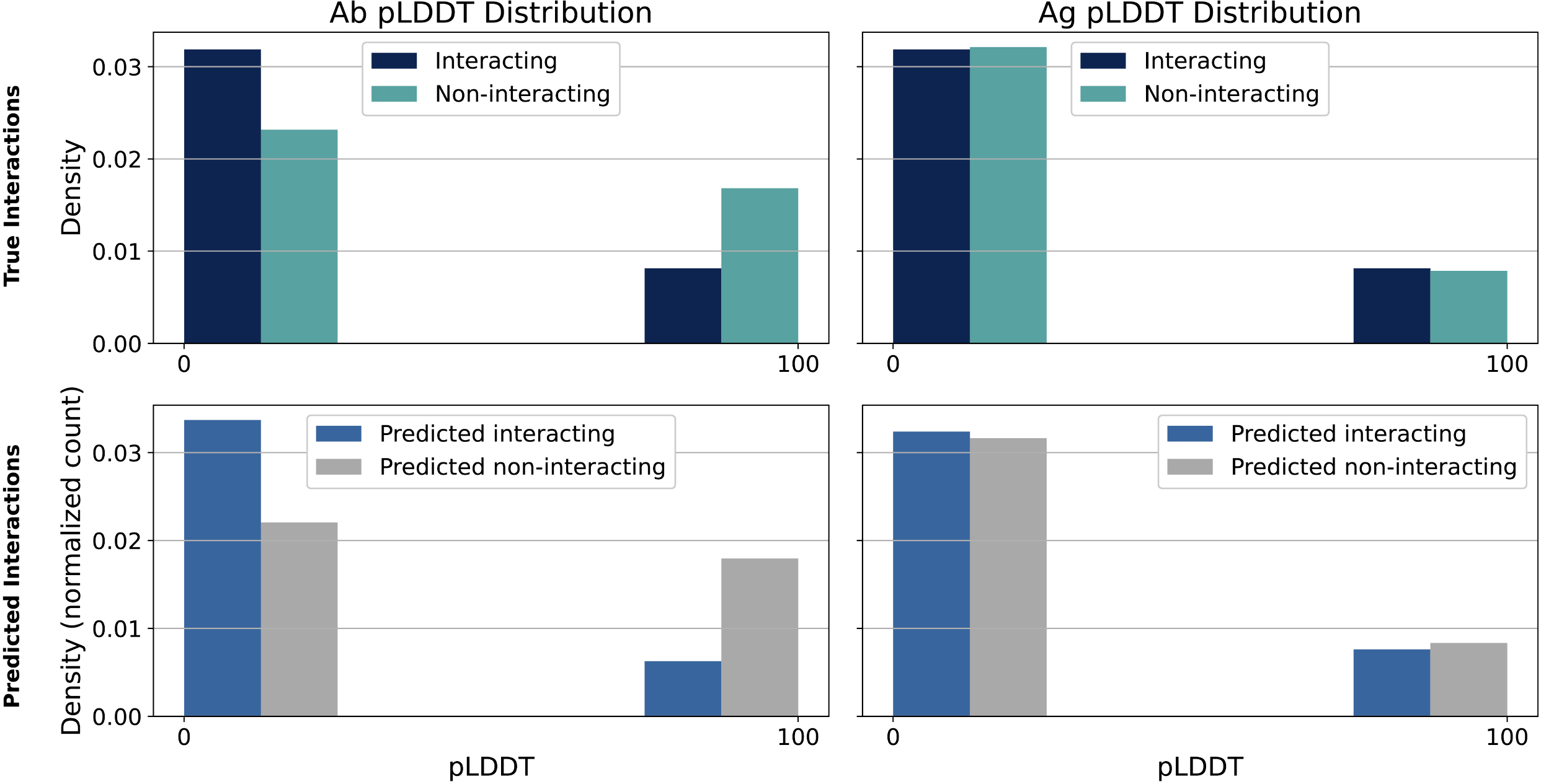

Supplement: S3 Fig — Top row: Normalized histograms of binary pLDDT values (0 = low confidence/flexible, 100 = high confidence/rigid) for ground-truth interacting (dark blue) and non-interacting (teal) residues. Antibody residues (left) show a higher density of interacting positions in low-pLDDT regions, consistent with expected flexibility of paratopes. In contrast, antigen residues (right) exhibit similar pLDDT distributions across both interacting and non-interacting sites. Bottom row: Analogous distributions based on predicted interactions from dMaSIF-search flex, with interacting (blue) and non-interacting (gray) categories. Predicted paratope residues also show greater enrichment in low-pLDDT regions, mirroring the ground truth trend. These results support the hypothesis that structural flexibility, as captured by low pLDDT, correlates with interaction propensity primarily on the antibody side. (TIFF) [file pcbi.1013576.s006.tif]

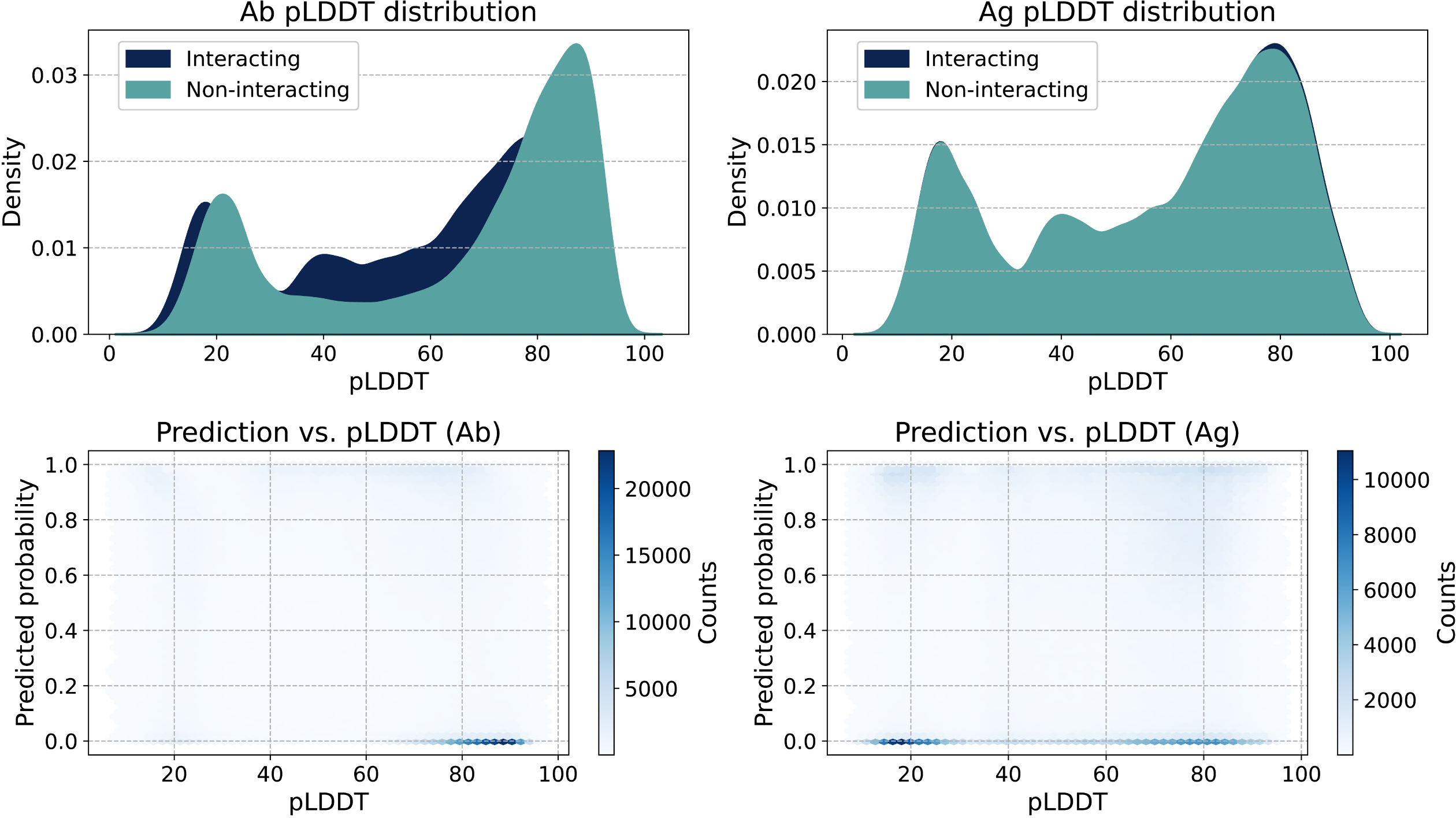

Supplement: S4 Fig — Top row: Density estimates of the pLDDT distributions for interacting (dark blue) and non-interacting (teal) points in antibodies (left) and antigens (right), based on ground truth labels. Antibody interaction sites show a moderate enrichment in low-pLDDT regions, consistent with increased flexibility at binding interfaces. In contrast, the pLDDT distributions of interacting and non-interacting antigen points are nearly indistinguishable. Bottom row: Hexbin scatter plots showing the relationship between predicted interaction probabilities (from dMaSIF-search flex) and pLDDT values for antibody (left) and antigen (right) points. Most high-pLDDT residues have low predicted interaction probabilities, suggesting that rigid regions are generally predicted as non-interacting. The pattern is more pronounced for antibodies, in line with the known flexibility of paratopes. Color bars indicate hexbin counts. (TIFF) [file pcbi.1013576.s007.tif]

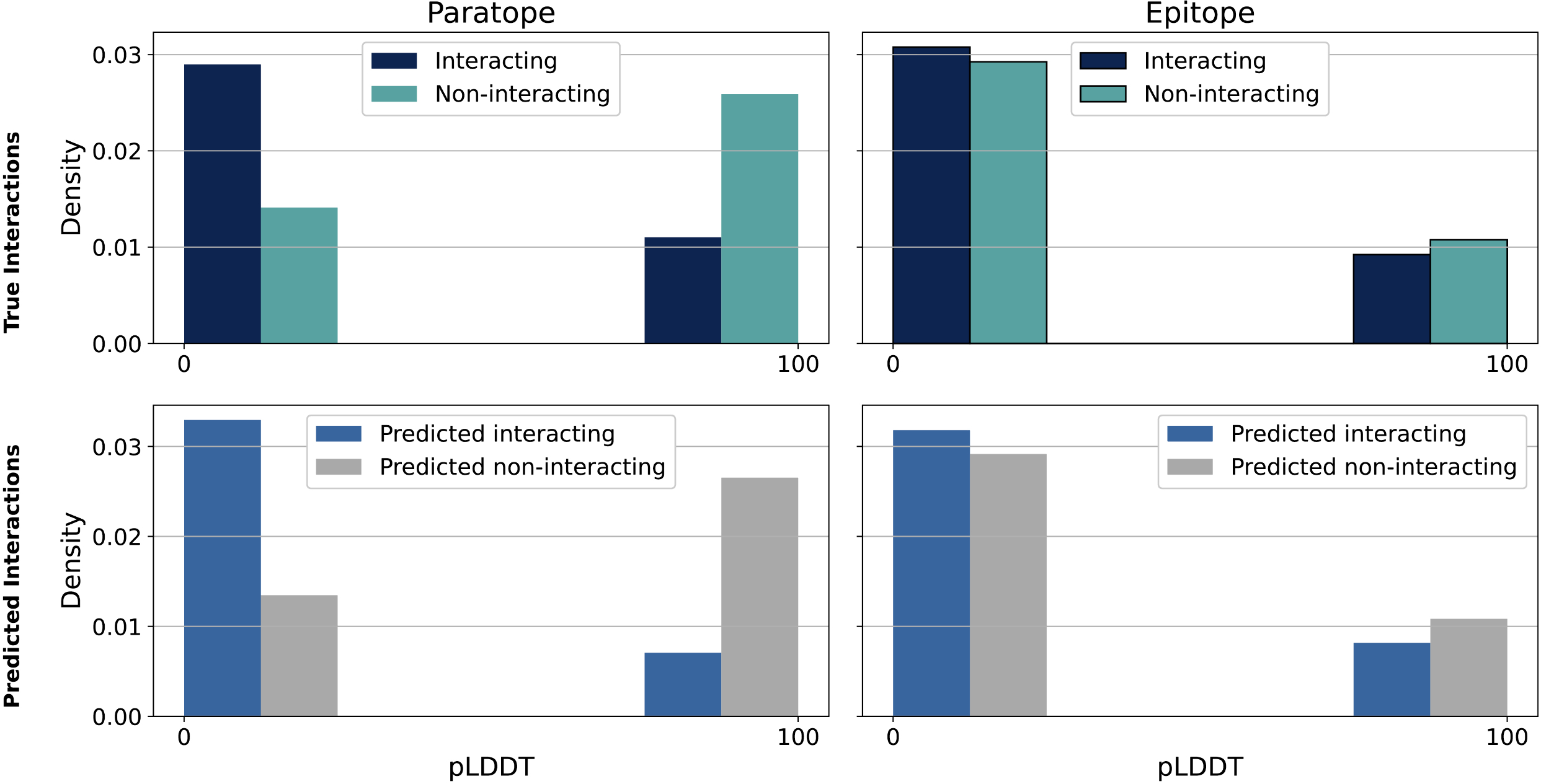

Supplement: S5 Fig — Top row: True interaction labels derived from antibody–antigen complex structures are used to separate residues into interacting (dark blue) and non-interacting (teal) classes. Distributions are shown for paratope (left) and epitope (right) residues. Interacting paratope residues are enriched at low-confidence (low pLDDT) regions, suggesting increased local flexibility near true binding sites. Bottom row: Predicted interactions from dMaSIF-site flex are used in place of true labels. Predicted interacting residues (blue) and predicted non-interacting residues (gray) show similar trends to the ground truth, with low-pLDDT enrichment in predicted paratopes and more uniform distributions in epitopes. (TIFF) [file pcbi.1013576.s008.tif]

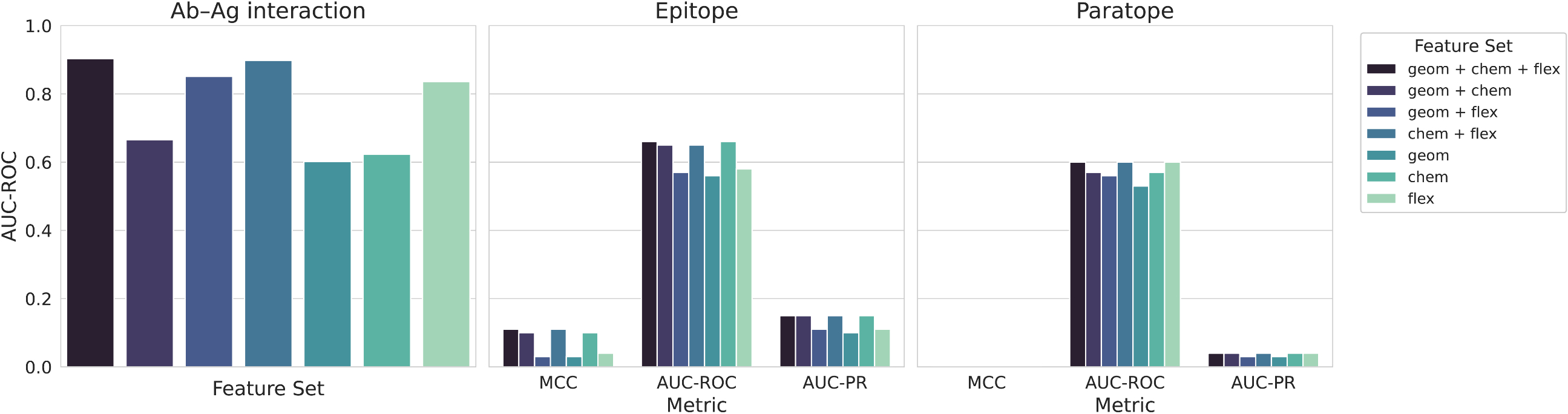

Supplement: S6 Fig — The bar plot illustrates how performance metrics (MCC, AUC-ROC, and AUC-PR) vary across different combinations of geometric ("geom"), chemical ("chem"), and flexibility ("flex") features. For Ab-Ag interaction predictions, flexibility and chemical + flexibility features are the most effective, highlighting the importance of using ITsFlexible representation in the model. In the case of epitope prediction, removing the flexibility feature has little impact, highlighting the stronger influence of chemical features. In contrast, for paratope prediction, flexibility, especially when combined with chemical information, proves to be the most impactful. Flexibility alone also shows a strong effect, suggesting that the observed drop in performance compared to pLDDT-based models can be largely attributed to the loss of this specific feature. (TIFF) [file pcbi.1013576.s009.tif]

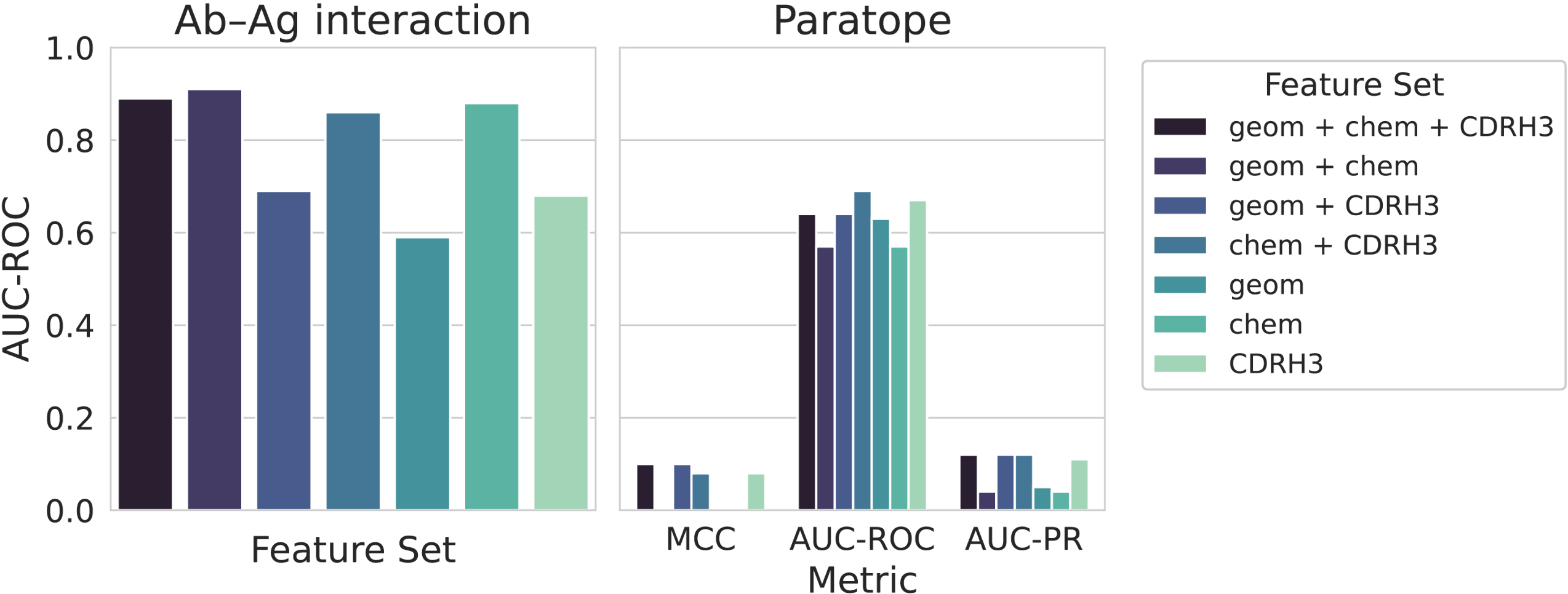

Supplement: S7 Fig — With CDRH3-only encodings, in the Ab–Ag interaction task the chemical features contribute most, and performance improves further when they are combined with geometric features. For paratope prediction, the CDRH3 one-hot channel becomes more informative, particularly in combination with chemical features. (TIFF) [file pcbi.1013576.s010.tif]

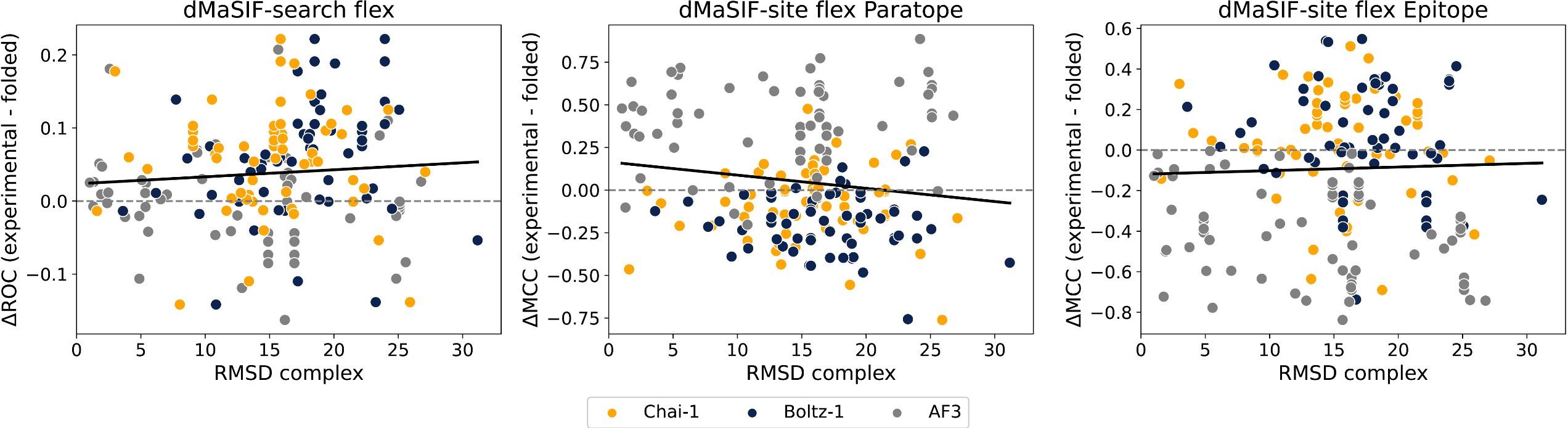

Supplement: S8 Fig — Each panel shows the change in prediction quality (Δ = experimental – refolded) versus the RMSD between predicted and crystal structures, across three refolding methods (Chai-1, Boltz-1, AF3). Left: Δ ROC AUC for the full complex shows a slight negative trend, suggesting that higher RMSD leads to marginally lower predictive quality. Middle: ΔMCC for the antibody interface shows a positive trend, indicating that higher complex RMSD improves paratope prediction because the interface is "compromised". Right: ΔMCC for the antigen interface mirrors the first panel, exhibiting a weak negative trend as RMSD increases, which reduces performance for epitope prediction. (TIFF) [file pcbi.1013576.s011.tif]
